# Oral Cavity Cancer Secondary to Dental Trauma: A Scoping Review

**DOI:** 10.3390/biomedicines12092024

**Published:** 2024-09-04

**Authors:** Carlos M. Chiesa-Estomba, Miguel Mayo-Yanez, Luigi A. Vaira, Antonino Maniaci, Allen L. Feng, Maria Landa-Garmendia, Adrian Cardin-Pereda, Jerome R. Lechien

**Affiliations:** 1 Otorhinolaryngology-Head & Neck Surgery Department, Donostia University Hospital, BioGuipuzkoa Research Institute, Faculty Of Medicine, Deusto University, 20014 Donostia, Spain; 2 Head & Neck Section—Research Committee of Young Otolaryngologists of the International Federation of Otorhinolaryngological Societies (IFOS), 13005 Marseille, France; 3 Department of Otorhinolaryngology-Head and Neck Surgery, Complexo Hospitalario Universitario de A Coruña (CHUAC), 15006 A Coruña, Spain; 4 Maxillofacial Surgery Operative Unit, Department of Medicine, Surgery and Pharmacy, University of Sassari, Viale San Pietro 43/B, 07100 Sassari, Italy; 5 Faculty of Medicine and Surgery, University of Enna “Kore”, 94100 Enna, Italy; 6 Department of Otolaryngology-Head and Neck Surgery, Massachusetts Eye and Ear, Boston, MA 02114, USA; 7 Maxillofacial Surgery Department, Donostia University Hospital, 20014 Donostia, Spain; 8 Division of Laryngology and Broncho-Esophagology, Department of Otolaryngology-Head Neck Surgery, EpiCURA Hospital, UMONS Research Institute for Health Sciences and Technology, University of Mons (UMons), 7000 Mons, Belgium

**Keywords:** oral cavity cancer, dental trauma, non-smoking patients, risk factors, carcinogenesis

## Abstract

(1) Background: Oral cavity cancer represents the most common site of origin of head and neck mucosal malignancies. A few limited studies have suggested that chronic irritation, particularly in non-healing ulcers, and fibrotic tissue from poor dentition or ill-fitting dentures had a role in developing mouth cancer. This scoping review aims to evaluate the existing evidence concerning Oral Cavicty Cancer (OCC) in non-smokers/non-drinkers and the relationship with dental trauma. (2) Methods: A scoping review of the PubMed, Embase, and Web of Science databases was completed in adherence with the Preferred Reporting Items for Systematic reviews and Meta-Analyses extension for Scoping Reviews checklist. (3) Results: Of the 33 articles that met inclusion, in 6 of them authors discussed the role of topography in dental trauma, in 11 articles authors discussed the carcinogenesis mechanism involved in chronic mucosal trauma, in 17 articles data on ill-fitting dentures was included, 4 studies dealt with the effect of broken/sharp teeth on mucosal damage, and in 7 studies the role of oral hygiene was covered. Less frequently discussed topics included gender, risk of neck nodes, and the role of potentially malignant oral disorders. (4) Conclusions: The available literature suggests a potential connection between chronic dental trauma and the development of OCC. Studies have highlighted factors such as denture use and ill-fitting dental appliances as contributors to an increased risk of oral cancer. Interestingly, we still miss data to support the hypothesis that women, particularly those without toxic habits like smoking or alcohol consumption, appear to be disproportionately affected by oral cancer related to chronic dental trauma.

## 1. Introduction

Oral cavity cancer (OCC) represents the most common site of origin of head and neck (H&N) mucosal malignancies [1]. These can arise from the buccal mucosa, alveolar ridges, lip, floor of the mouth, retromolar trigone, oral tongue, and hard palate, with an estimated worldwide incidence of approximately 377,000 cases in 2020 according to GLOBOCAN [2].

Epidemiologically, OCC is commonly associated with smoking, alcohol consumption, chewing of betel nut, spices, and untreated syphilis in developing countries such as India and Pakistan, among others [3,4]. However, a few limited studies have suggested that chronic irritation, particularly in non-healing ulcers and fibrotic tissue [5,6], from poor dentition or ill-fitting dentures had a role in developing mouth cancer, especially in non-smoking female patients [7,8,9]. This was recognized in 1863 by Rudolf Virchow, who postulated that chronic irritation and previous injuries are a precondition for tumorigenesis [5].

In the oral cavity, as mentioned above, chronic irritation may result from poor oral hygiene [10], poor dentition [11], missing teeth [12], and ill-fitting dentures [13]. Moreover, in a review published by Llewellyn et al. on potential risk factors for OCC among young adults, the authors revealed a lack of specific research in this topic, highlighting the potential carcinogenic effect of different factors, including viruses, the oral microbiome, marijuana use, and dental health practices [14]. Increasing evidence on genome sequencing, like TP53 and CDKN2a mutations, has revealed a potentially unique genetic landscape for OCC in non-smoking patients. However, further research is still needed to uniformly elucidate the diversity of oncogenic drivers [15,16,17,18,19].

According to the literature, 30% to 35% of all H and N mucosal malignant neoplasms arise in the oral cavity [19]. In contrast, according to Wiseman et al., in the non-smoking population, up to 75% of all H and N mucosal malignancies affect the oral cavity [20]. This evidence suggests the potential role of chronic trauma in the development of OCC in non-smoking patients. However, recurrent chronic trauma is still not widely accepted as a carcinogen. For this reason, H and N surgeons and dentists might pay little attention to chronic teeth trauma or denture irritation as causative factors of premalignant lesions and OCC. Moreover, clinicians rarely document if dental trauma was present before the development of symptoms, even considering that chronic inflammation and the subsequent oxidative stress has been associated with carcinogenesis [21].

Standard of care for OCC still involves surgery for both locoregionally confined and advanced disease, with concurrent elective neck dissection in node-positive necks or based on tumor depth seeking to remove subclinical regional lymph node metastasis [1,22]. Primary radiotherapy is reserved as an option for locally advanced OCC and unresectable disease, given an improved prognosis of primary resection when feasible [23,24,25]. With the considerable comorbidities associated with treatment of OCC and overall heterogeneity in outcomes, it is advisable to compile data concerning the risk of OCC in non-smoking and non-drinking patients, and to investigate whether this represents a distinct disease entity.

The specific research question is: ‘Is dental trauma a carcinogenic factor for oral cavity cancer in non-smoking and non-drinking patients?’

## 2. Materials and Methods

**Figure 1:**
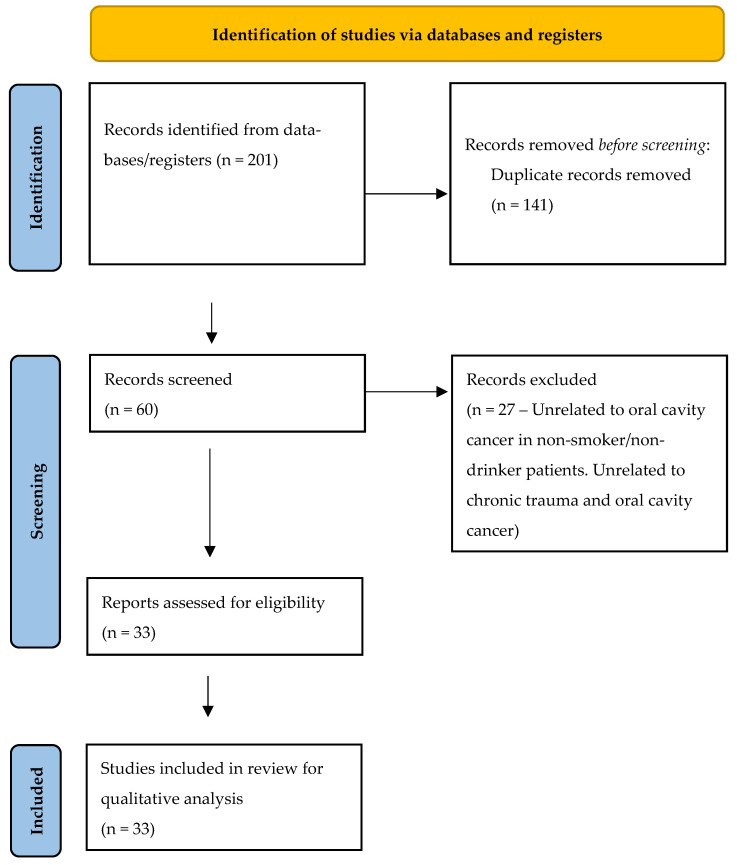
PRISMA Flow Diagram.

A scoping review of the PubMed, Embase, and Web of Science databases was completed in adherence with the Preferred Reporting Items for Systematic reviews and Meta-Analyses extension for Scoping Reviews (PRISMAScR) checklist (Figure 1) (Supplementary Material). The study protocol was uploaded onto the Open Science Framework (https://osf.io/3h2b7) on 12 June 2024. The search strategy was performed in using related terms ‘(“oral cavity” [MeSH Terms] OR “oral cavity” [All Fields]) AND (“non-smoker” [All Fields] AND “and” [All Fields] AND “non-smoker” [All Fields]) OR “non-smoker” [All Fields] OR (“non-smoker” [MeSH Terms] OR “squamous cell carcinoma” [All Fields] OR (“squamous cell carcinoma” [MeSH Terms] OR “squamous cell carcinoma” [All Fields]) AND ‘(“Dental Trauma” [MeSH Terms] OR “Dental Trauma” [All Fields]) AND (“Dental Trauma” [All Fields].’ AND (“non-drinker” [All Fields] AND “and” [All Fields] AND “non-drinker” [All Fields]) OR “non-drinker” [All Fields] OR (“non-drinker” [MeSH Terms]. A ‘snowball’ search was also performed with the references of the identified studies. The search strategy included studies between January the 1st 1988 and March the 1st 2024. Two independent reviewers (C.CH and M.M) performed the search. We searched all primary references for studies of oral cavity cancer secondary dental trauma, chronic trauma and studies of OCC in non-smokers and non-drinker patients. We did not exclude content based on publication year and only included publications in the English language.

We examined the full texts of the selected manuscripts, and the included types of studies were meta-analyses and systematic reviews, clinical trials, case series, and prospective and retrospective cohort studies published in peer-reviewed journals. No case reports, theses, grey literature, letters to editor, commentaries or meeting communications were included. There were no restrictions by date of publication.

Selection differences were resolved by consensus of senior authors. We considered studies that included information about smoking status in adult patients with oral cavity cancer or information about dental trauma or prosthesis trebling and who underwent surgical on non-surgical treatment and reported local survival outcomes. Excel (Microsoft Corp., Redmond, WA, USA) spreadsheets were used to capture study data. A proper meta-analysis was not performed due to the heterogeneity of data available.

## 3. Results

The literature search performed yielded 60 manuscripts after deduplication. Following title/abstract screening and full-text review, 33 articles met criteria for inclusion and were included in final data analysis. Of the 33 articles that met inclusion, in 6 of them authors discussed the role of the topography of dental trauma, in 11 articles authors discussed the carcinogenesis mechanism involved in chronic mucosal trauma, in 17 data about ill-fitting dentures were discusses, 4 dealt with the effect of broken/sharp teeth over mucosal damage, and in 7 studies the role of oral hygiene was covered. Less frequently discussed topics included gender, risk of neck nodes, and the role of oral potentially malignant disorders (Table 1).

## 4. Discussion

### 4.1. Which Is the Most Common Topography of Dental Trauma?

In general, the most common site for oral cavity cancer (OCC) occurrence is the lateral border of the tongue, observed in both smokers and non-smokers [35]. This finding suggests that the lateral border of the tongue could be a frequent site for chronic dental trauma. Clinical evidence also indicates an association between chronic mucosal trauma and oral cancer, primarily linked to tongue neoplasms, with a ninefold increase [8,9]. However, a study by Kruse et al., examining 67 oral cancer cases in non-smokers and non-alcoholic patients, found that the majority of OCC cases were located on the alveolar ridge [26]. A retrospective analysis by Perry et al., involving 390 patients with oral cancer, compared the most common sites of OCC among smokers and non-smokers. The authors found that OCC predominantly occurs at sites susceptible to dental and denture trauma, particularly the lateral edge of the tongue, especially in non-smokers without other risk factors. The occurrence of cancer in this anatomical subsite has a sixfold higher incidence than in the floor of the mouth in non-smokers, suggesting the potential role of teeth irritation as a carcinogen [35].

Dahlstrom et al. studied a group of non-smoker/non-drinker OCC patients over a period of ten years and found a bimodal distribution regarding age and tumor location, with peaks in the fifth and eighth decades of life. Younger patients primarily experienced mobile oral tongue involvement, while older patients (>70 years) had buccal/gingival mucosa as the most common subsite [27]. Similar results were published by Yesensky et al. [28].

Recent data suggest that the location of oral cavity cancers differs between smokers and non-smokers, with a higher proportion occurring on the edge of the tongue rather than in the floor of the mouth, affecting young non-smokers due to the range of sharp teeth or rough dental edges. The gingiva, floor of the mouth, and buccal mucosa are identified as key sites of trauma from chronic denture rubbing [35].

### 4.2. What Is the Current Knowledge about the Carcinogenesis Mechanism in Chronic Mucosal Trauma?

**Figure 2:**
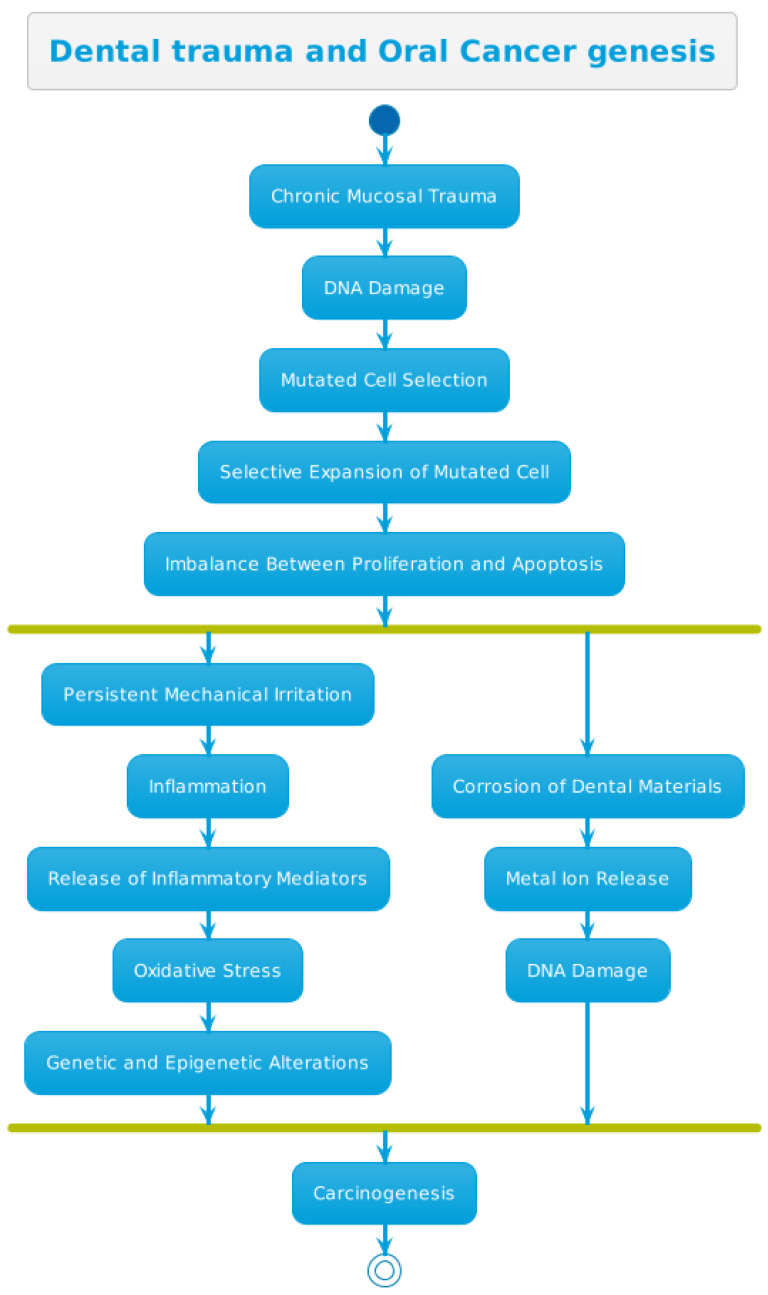
Pathogenic process diagram linking dental trauma to the genesis of oral cancer.

Carcinogenesis represents a multistep process that begins with the selection of a mutated cell, followed by the selective expansion of this cell and progression due to an imbalance between cellular proliferation and death (Figure 2).

Throughout this process, numerous genetic and epigenetic events act as drivers, transforming normal cells into malignant tumors and conferring various types of growth advantages [21].

Experimental animal studies have suggested that chronic trauma may lead to cancer formation through two different mechanisms. Firstly, persistent mechanical irritation can cause DNA damage, eventually resulting in cancer formation. This has been evidenced by increased activity of poly-ADP-ribose polymerase in patients with chronic mucosal trauma [36]. In fact, a wide array of infectious and non-infectious chronic inflammatory conditions can predispose susceptible cells to neoplastic transformation [37].

The longer inflammation persists, the higher the risk of cancer development, as inflammation alters the injury, repair, and resolution processes. The accumulative effect of inflammatory cells (neutrophils, monocytes, macrophages, eosinophils, dendritic cells, mast cells, and lymphocytes) may contribute to the onset and progression of cancer. Additionally, there is upregulation of prostaglandins, cytokines, nuclear factor NFkB, chemokines, angiogenic factors, and the release of free radical species derived from oxygen (ROI) and nitrogen (RNI) [21].

The second theory proposes that chronic mucosal trauma can trigger inflammation, followed by the release of chemical mediators, such as cytokines, prostaglandins, and tumor necrosis factor, which in turn lead to oxidative stress [38]. These changes can induce genetic and epigenetic alterations, damaging DNA, affecting its repair capability, altering transcription factors, preventing apoptosis, and stimulating angiogenesis, ultimately resulting in carcinogenesis. In summary, inflammation may act at various stages, leading to cancer formation [39,40].

Despite chronic trauma being considered as the primary carcinogenic mechanism, evidence suggests a potential correlation between denture material and the risk of OCC. The presence of certain monomers in denture prostheses is considered a crucial part of chronic irritation for oral mucosa [41,42,43,44]. In this regard, in vivo and in vitro studies have demonstrated that saliva can act as an electrolyte solution for metals, increasing corrosion, promoting the liberation of ions, altering local pH, and generating enzymatic reactions with mutagenic effects in the oral cavity mucosa [45,46].

Regarding the different metals used in dental material fabrication (such as brackets, wires for braces, retainers, dentures, implants, posts, and crowns), stainless steel, cobalt–chromium alloys, titanium metal, titanium, or precious metals (gold, platinum, palladium, or silver) may contain smaller amounts of many other added elements, such as mercury, to improve the alloys’ properties [47]. Evidence from metal toxicology indicates the carcinogenic properties of metal ions due to DNA damage, including nickel and cobalt–chromium, which are significant components of orthodontic and dental appliances [48,49,50]. Studies have shown significant differences in DNA damage and chromium content in buccal mucosal cell samples collected before and after orthodontic appliance placement, highlighting the potential risks associated with these materials [45,51]. Additionally, the use of dentures containing metal has been associated with a greater risk of oral cancer, although no relationship has been found with the use of acrylic dentures [11].

Another potential subset for malignant development comes from the group of oral potentially malignant disorders (OPMDs), although these have been scarcely evaluated. A retrospective study demonstrated a significant association between OCC and chronic mucosal trauma, but no such association was confirmed between OPMD and mucosal trauma [11].

Although the carcinogenic effect of dental implants has never been conclusively established, some theories suggest that the release of corrosion products, metallic ion release, and migration of malignant cells around the implant may contribute to carcinogenesis [52,53]. In a systematic review, 75% of dental implant-associated OCC cases had a previous history of cancer, emphasizing the importance of checking for ill-fitting fixed dentures in contact with altered mucosa due to toxic habits, in order to mitigate the increased risk of developing OCC [54].

In a study by Yesensky et al., a high prevalence of orthodontic treatment was found in younger patients within 15 years of the original cancer diagnosis, indicating a need for further investigation into the potential link between metallic orthodontic hardware and subsequent development of OCC. Additionally, a high frequency of metallic dental hardware was identified in OCC patients older than 50 years before their cancer diagnosis, with 82% of patients in this subgroup using crowns/bridges, dental implants, and/or dentures with metallic elements [28].

### 4.3. Is a Role of Ill-Ftting Dentures in Oral Cavity Cancer Development?

There are many studies in the literature that have proven the association between dentures and chronic mucosal damage due to chronic mucosal insult and subsequent cancer formation. However, the evidence against or in favor of this is sometimes contradictory. Factors like the type of denture, the time of use, or the influence of oral hygiene, among others, have been studied.

There are crucial steps during the wound healing process. All of them involve some dynamic interactions between cells, leading to rapid wound closure and subsequent tissue repair, and will act as a cascade of events, including inflammation, followed by fibroblast proliferation, remodeling, angiogenesis and the deposition of new connective tissue, which is known as granulation tissue [55,56]. According to Thumfart et al. in a study of OCSCC, in 44% of the patients included, the authors found a correlation between the site of cancer development and the presence of chronic dental irritation. However, is it important to highlight that the authors do not separate patients regarding smoking status [30]. In another Swedish epidemiological study, [7] the authors found that the relative risk for oral cancer was six times higher in painful dentures and three times higher in patients with ill-fitting dentures, and also more common in females than in males. Again, in this study, authors did not separate patients according to their smoking status.

A meta-analysis published by Manoharan et al. [13], which investigates the correlation between dentures and OCSCC, demonstrates that the use of dentures by itself was associated with an increased risk of developing cancer (OR = 1.42, 95% CI = 1.01–1.99), while ill-fitting dentures increase the risk of developing cancer by almost four times (pooled OR = 3.90, 95% CI = 2.48–6.13)

By contrast, another study published by Velly et al. [8] reported that patients with prolonged use of dentures (>8 years) experienced a lower risk of tongue cancer. In this study, authors hypothesized that this may be related to the use of well-fitted dentures. They also suggest that patients with a regular follow-up by health-care personnel may have a lower risk of chronic trauma. In the same vein, in the meta-analysis published by Manoharan et al. [13], in which patients were classified as long-term users (>5 years) or short-term (<5 years), the authors showed that there was no link between the duration of denture use and cancer development.

Retrospective data about the role of oral hygiene, dental condition, diet, and nutrition and their influence on OCC development has been published [28]. Moreover, results published by Rosenquist showed that defective or malfunctioning complete dentures was a significant risk factor for the development of OCSCC, being higher in those patients with poor oral hygiene [31].

Other authors such as Vacarrezza et al. have found an association between ill-fitting dentures and OCC. They hypothesized that chronic physical irritation of the oral mucosa contributes to the topical carcinogenic effect of tobacco [30]. The same results were reported by Bundgaard et al.; however, when they adjusted the risk for associated factors, such as tobacco, alcohol, and dental-related factors (broken tooth, decayed teeth, filled teeth), they did not find a significant correlation [32]. On the other hand, some studies found a correlation between teeth status and OCC risk, suggesting tooth loss as one of the most important dental factors related to OCC. However, they did not find a significant risk between denture use and OCC development [33,57].

Some authors also mention the potential correlation between some chronic colonization of dentures and OCC. A recent study published by Jainkittivong et al. demonstrated the influence of candida colonization around the denture-based materials and the induced inflammation of the mucosa [58], a factor correlated with the development of epithelial dysplasia and leukoplakia formation [59]. Both lesions are well recognized as oral premalignant lesions with a higher potential for malignancy [60]. Another Japanese retrospective study [61] demonstrated the presence of human papillomavirus (HPV) in denture wearers. The authors of this study hypothesized that dentures could serve as a reservoir of HPV from different types of HPV-associated diseases.

### 4.4. Is There a Role for Broken/Sharp Teeth in Oral Cavity Cancer Development?

While there are no specific studies directly addressing the association between sharp teeth and oral cavity cancer (OCC) development, several studies have explored the relationship between defective teeth and OCC risk.

A study conducted by Rosenquist revealed that patients with five or more defective teeth have a threefold higher risk of developing OCC [31]. However, a limitation of this study was the lack of specific criteria to define the concept of defective teeth. In contrast, a study by Velly et al. found that OCC risk was lower in patients with broken teeth but without toxic habits. This suggests a nuanced relationship between dental conditions and OCC risk [8].

On the other hand, Marshall and Young did not find any correlation between broken teeth and OCC in a study where patients were not separated according to their smoking status. This discrepancy underscores the need for further research to elucidate the precise role of dental conditions, including sharp teeth, in OCC development [12,29].

What is the influence of gender and age on oral cavity cancer in non-smokers/non-alcoholic patients?

A retrospective analysis conducted by Perry et al. revealed that, among non-smokers and non-tobacco chewers with oral cancer, the majority were women, comprising over 60% of patients in their study [35]. This finding aligns with another descriptive study by Albuquerque et al., which observed differences in sex distribution based on smoking and alcohol status. In their study, 26% of females did not have toxic habits compared to only 11% of males [34]. This correlation is supported by other studies highlighting a higher incidence of OCC in women without toxic habits. However, a common limitation in these studies is the lack of data regarding the association between dental or denture-related irritation and the appearance of OCC.

In the study by Dahlstrom et al., a bimodal distribution of age at presentation was observed among women without toxic habits, with tongue cancers being more common in the fifth decade of life and gingivo-buccal cancers in the eighth decade [27]. These results are consistent with those of Perry et al., where the mean ages of non-smoking patients with alveolar arch tumors was 75 years and 67 years for patients with floor of mouth tumors, while the mean age of patients with lateral tongue cancer was 62 years [35]. The authors hypothesized that younger women were more likely to have their own teeth, which could potentially break or become rough medially. In contrast, older patients in their 80s would be more likely to have full dentures. Generally, acrylic or porcelain dental crowns are well-aligned to prevent rubbing against the tongue. However, in patients with long-standing dentures, the undersurface can cause trauma to the alveolus, floor of the mouth, and lower buccal mucosa.

### 4.5. Are Metastatic Neck Nodes Common in Patients with Oral Cavity Cancer Secondary to Chronic Trauma?

We came across a retrospective study that explores the correlation between dental factors, oral cavity cancer (OCC), and neck nodes [8]. Interestingly, the study found that patients with primaries adjacent to teeth and dental appliances tend to have a lower number of neck nodes affected. The authors propose that associated symptoms, such as pain, bleeding, or functional issues, prompt these patients to seek dental care earlier, potentially leading to earlier detection and management of OCC. However, it is important to note that there is currently no specific study or evidence regarding nodal disease in OCC patients with tumors secondary to chronic trauma.

### 4.6. What Is the Effect of Oral Hygiene on the Development of Oral Cavity Cancer?

One known risk factor for the emergence of oral cavity cancer (OCC) is poor dental hygiene. Numerous investigations have looked into the connection between oral hygiene habits and the likelihood of OCC. Talamini et al. conducted a retrospective study to examine the effects of nutrition, food, dental health, and oral hygiene on a cohort of more than 400 OCC patients. The risk of developing OCC was shown to be strongly correlated with how often teeth were brushed. Compared to patients who brushed more regularly, those who reported brushing less than once a day had a considerably increased chance of acquiring OCC [57]. Rosenquist et al. published similar findings, indicating that teeth brushing infrequently was a substantial risk factor for the development of oral cancer (OCC). The risk of malignant transformation may increase as a result of persistent inflammation and carcinogen accumulation in the oral cavity, according to the author’s hypothesis [31]. This is in line with recent research that has connected persistent inflammation to the emergence of several malignancies, including OCC [62,63]. However, after controlling for confounding variables, including alcohol and tobacco use, research by Velly et al. revealed no significant correlation between OCC and the use of dentures, fractured teeth, or other dental problems [8]. This implies that there may be a more nuanced association between dental disorders and the risk of OCC that is influenced by other lifestyle factors and opens the way for future research into factors such as the oral microbiome.

More recently, the role of oral microbiome changes and OCC has been studied. According to Yan et al., the presence of Fusobacterium, Peptostreptococcus, Neisseria, and Parvimonas were increased in cancer compared to controls, and particularly increased in advanced-stage cancers compared to early-stage disease. They also found an inverse correlation with normal flora of the oral cavity, with a significant decrease in Streptococcus, Rothia, Actinomyces, and Megasphaera. As a conclusion of this study, the authors suggest that these bacteria can be employed as a potential marker for tumor progression or can be interrogated to better characterize the tumor microenvironment [64].

Finally, a recent systematic review and meta-analysis on the relationship between periodontitis and OCC revealed significant yet cautiously interpreted findings [65]. Ma et al. analyzed 16 studies involving 6032 OCC patients and 7432 healthy controls. The meta-analysis, based on data from nine studies, showed a significant correlation between periodontitis and the risk of OCC (OR = 2.94, 95% CI: 2.13–4.07), although the evidence was of low certainty. Additionally, individuals with more than 15 missing teeth had a higher risk of OCC (OR = 1.91, 95% CI: 1.01–3.62). It was also observed that clinical attachment loss (CAL) and the decayed, missing, and filled teeth index (DMFT) were more pronounced in OCC patients compared to the control group (CAL, SMD = 1.94, 95% CI: 0.22–3.66; DMFT, SMD = 0.65, 95% CI: 0.12–1.18). They concluded that periodontitis may serve as a potential risk factor for OCC; however, these findings should be interpreted with caution due to the substantial level of heterogeneity present in the analyzed studies.

It is crucial to remember that the majority of the research that is now accessible has a retrospective design, which is vulnerable to recall bias and might not fully reflect the long-term effects of oral hygiene habits on the risk of OCC. Furthermore, the examination of dental diseases and oral hygiene has been quite rudimentary, frequently depending on self-reported information or scant clinical assessments. In the future, research should use prospective designs with more thorough and objective assessments of dental condition, oral hygiene, and other pertinent characteristics to better understand the role of oral hygiene in OCC development. This will direct the creation of focused preventive initiatives and assist in identifying the precise processes by which poor oral hygiene may increase the risk of OCC. Based on available data, there may be a connection between oral hygiene issues and a higher chance of oral cancer. However, more research through carefully planned, prospective trials is necessary to determine the precise nature of this association and the underlying mechanisms. Enhancing our knowledge of how oral hygiene affects the development of OCC may have significant effects on cancer prevention and early detection programs that target high-risk groups.

### 4.7. What Are the Current Clinical Limitations?

The available literature lacks information regarding the morphology of tumors associated with chronic dental trauma. Additionally, the recurrence rate, outcomes, and prognosis of such tumors have not been studied separately. Most of the existing literature comprises retrospective case-control and descriptive studies. These studies have inherent limitations, including recall bias, selection bias, nonblinding, and over- or under-matching. Therefore, OCC patients without toxic habits represent a growing population, and there is a need to determine if special consideration is needed to optimize treatment modalities. Authors, like Brennan et al., suggest that immunotherapy may be useful for these patients due to the higher PD-L1 expression [66].

Despite these limitations, case-control studies suggest a potential role played by factors associated with chronic mucosal trauma in the etiology of oral cancer. Further research, ideally prospective and with larger sample sizes, is needed to better understand the relationship between chronic dental trauma and oral cancer development, recurrence, and prognosis.

### 4.8. Future Directions

The available literature suggests a potential connection between chronic dental trauma and the development of OCC. Studies have highlighted factors such as denture use and ill-fitting dental appliances as contributors to an increased risk of oral cancer. Interestingly, we still miss data to support the hypothesis regarding women, particularly those without toxic habits, like smoking or alcohol consumption, who appear to be disproportionately affected by oral cancer related to chronic dental trauma.

Moreover, age distribution patterns in OCC cases associated with chronic dental trauma show a bimodal distribution. Younger patients tend to present with tongue cancers, while gingivo-buccal cancers are more common among older patients. This variation may be linked to differences in dental status and the duration of denture use.

However, despite these observed associations, there is a notable absence of specific studies focusing on the morphology of tumors associated with chronic dental trauma, recurrence rates, outcomes, and prognosis. The existing literature primarily consists of retrospective case-control or descriptive studies, which come with inherent limitations, such as recall bias, selection bias, and lack of blinding. This underscores the need for further research, particularly prospective studies with larger sample sizes, to provide more robust evidence.

## 5. Conclusions

While suggestive evidence exists for a link between chronic dental trauma and oral cancer, there is still much to be understood about the exact mechanisms involved, the specific characteristics of associated tumors, and the long-term outcomes for affected individuals.

## Figures and Tables

**Table 1:** Summary of studies included. Abbreviations: Oral Cavity Cancer (OCC).

Authors/Year/Country	Study Design	Objective	Patients Characteristics	Subsites/Location	Conclusion
Perry et al.—2015—USA [26]	Retrospective	Determine whether OCC occurred more commonly at sites of dental trauma and how the position of these cancers varied between non-smokers	Smokers: 390 Non-Smokers: 87	Buccal mucosa, alveolar ridge, floor of the mouth, lateral tongue, dorsum of the tongue, retromolar trigone.	OCC occurs predominantly at sites of potential dental and denture trauma, especially in non-smokers without other risk factors.
Velly et al.—1998—Brasil [8]	Case-Control	Examine the association between dental health characteristics and the risk of OCC.	306 patients.	Tongue, floor of the mouth, gum, others.	Poor oral hygiene due to infrequent tooth brushing and sores caused by dentures are risk factors for OCC
Lockhart et al.—1998—USA [9]	Prospective	Investigates the potential role of dental and other local factors in the genesis of OCC.	28 patients with oral cancer	Retromolar trigone, mandibular ridge, lateral border of the tongue, anterior or lateral floor of the mouth.	Authors’ findings suggest a potential relantionshipt between dental factors and the genesis of OCC.
Kruse et al.—2010—Switzerland [26]	Retrospective	Evaluate the clinicopathologic characteristics of OCC in non-smokers and non-drinkers.	67 patients	Retromolar trigone, buccal mucosa, maxilla, alveolar ridge, floor of the mouth, tongue.	Authors found a tendency toward a higher proportion of female patients over 70 years and a higher number of OCCs.
Dahlstrom et al.—2008—USA [27]	Prospective	Tries to define the demographics and potential risk factors of OCC in non-smokers/non-drinkers patients	172 non-smokers/non-drinker patients	Oral tongue, gingiva, buccal mucosa, retromolar trigone, floor of the mouth, hard palate.	Non-smokers/non-drinkers with OCC are commonly young women with oral tongue cancer and elderly women with gingival/buccal cancer
Yesensky et al.—2018—USA [28]	Retrospective	Investigates the role of exposure to dental hardware inOCC.	54 patients	Tongue, alveolar ridge, gingiva, buccal mucosa, retromolar trigone, hard palate.	Exposure to metallic dental hardware has increased recently given the rise of orthodontic braces and older adults retaining more teeth. Despite that authors did not find a causal relationship between OCC and dental hardware, this is a step towards investigating its role.
Thumfart et al. —1978—Germany [7]	Retrospective	Identifies the role of mechanical trauma in OCC etiology	86 patients	Tongue, floor of the mouth, palate, gum, cheeks.	The permanent traumatization of the covering squamous epithelium was in correlation with all forms of inflammation, but also parakeraosis and hyperkeratosis, dysplasia, including carcinoma in situ, and manifest carcinomas.
Young et al.—1986—USA [29]	Case-Control	Explores the characteristics of oral cancer patients and potential carcinogenic exposure in the oral cavity (dentures)	202 patients	Not described	Authors found a correlation between the site of cancer development and chronic dental irritation.
Vaccarezza et al.—2010—Brasil [30]	Case-Control	Examines whether denture use and recurrent sores caused by ill-fitting dentures are associated with OCC in individuals exposed to tobacco.	Not described	Not described	The association between recurrent oral sores caused by ill-fitting dentures and squamous cell carcinoma of the mouth in smokers is in agreement with the hypothesis that the chronic physical irritation of oral mucosa contributes to the topical carcinogenic effect of tobacco
Rosenquist et al.—2005—Sweden [31]	Case-Control	The aims of the study were to assess different potential risk factors in OCC such as oral hygiene, dental status, oral mucosal lesions, alcohol and tobacco use, virus infection, and those related to lifestyle.	Not described	Not described	Independent risk factors identified were poor oral hygiene, inadequate dental status and malfunctioning complete dentures.
Bundgaard et al.—1995—Denmark [32]	Case-Control	Evaluates risk of OCC development.	161 patients	Not described	Denta status was confirmed as a potential risk for OCC development.
Schildt et al.—1998—Sweden [33]	Case-Control	Authors investigated the role of oral infections, dentition and dental X-rays in OCC development	410 patients	Not described	A multivariate analysis suggested that the most important risk factors were oral infections followed by liquor consumption and active smoking
Alburqueque et al.—2011—Portugal [34]	Retrospective	The aim of this study was to determine the relationship between the use of removable dentures and the presence of squamous cell carcinoma in the anterior two thirds of the tongue	106 patients	Not described	The authors did not find a relationship between denture use and the presence of OCC in the two anterior thirds of the tongue

## Data Availability

For this study, no new data were created.

## Supplementary Materials

The following supporting information can be downloaded at: https://www.mdpi.com/article/10.3390/biomedicines12092024/s1; File S1: Preferred Reporting Items for Systematic reviews and Meta-Analyses extension for Scoping Reviews (PRISMA-ScR) Checklist.
